# Country contextualisation of cost-effectiveness studies: lessons from Ethiopia

**DOI:** 10.1136/bmjgh-2018-001320

**Published:** 2019-12-01

**Authors:** Kjell Arne Johansson, Mieraf Taddesse Tolla, Solomon Tessema Memirie, Ingrid Miljeteig, Mahlet Kifle Habtemariam, Addis Tamire Woldemariam, Stéphane Verguet, Ole Frithjof Norheim

**Affiliations:** 1 Department of Global Public Health and Primary Care, University of Bergen, Bergen, Hordaland, Norway; 2 Department of Global Health and Population, Harvard T.H. Chan School of Public Health, Boston, Massachusetts, USA; 3 Department of Public Health, Addis Ababa University, Addis Ababa, Oromia, Ethiopia; 4 Department of Research and Development, Haukeland University Hospital, Bergen, Norway; 5 Federal Ministry of Health, Addis Ababa, Ethiopia

**Keywords:** cost-effectiveness analysis, essential health services, universal health coverage, priority setting, ethics

## Abstract

Emerging demographic, epidemiological and health system changes in low-income countries require revisions of national essential health services packages in accordance with standard healthcare priority setting methods. Policy makers are in need of explicit and user-friendly methods to compare impact of multiple interventions. We provide experiences of country contextualisation of WHO-CHOICE methods and models to a country level. Results from three contextualised cost-effectiveness analyses (CEAs) are presented, and we discuss how this evidence can inform priority setting in Ethiopia. Existing models for a range of interventions in obstetric and neonatal care, psychiatric and neurological treatment and prevention and treatment of cardiovascular diseases are contextualised to the Ethiopian setting. CEAs are defined as contextualised if they include national analysts and use country-specific input for either costs, epidemiology, demography, baseline coverage or effects. Interventions (n=61) are ranked according to incremental cost-effectiveness rates (ICERs), and expected health outcomes (Disability Adjusted Life Years (DALYs) averted) and budget impacts are presented for each intervention. Dominated interventions (n=30) were excluded. A US$2.8 increase per capita in the annual health budget is needed in Ethiopia (currently at US$28 per capita) for increasing coverage by 20%–75% for all the 22 interventions with positive net health benefits. This investment is expected to give a net benefit at around 0.5 million DALYs averted in return in total, with a willingness to pay threshold at US$2000 per DALY averted. In particular, three interventions, neonatal resuscitation, kangaroo mother care and antibiotics for newborn sepsis, stand out as best buys in an Ethiopian setting. Our method of contextualised CEAs provides important information for policy makers. Rank ordering of interventions by ICERs, together with presentations of expected budget impact and net health benefits, is a clear and policy friendly illustration of possible efficient stepwise pathways towards universal health coverage.

**Summary box**

- Health economic evaluations and contextualised cost-effectiveness analyses (CEAs) are valuable tools for priority setting in emerging demographic, epidemiological and health system changes in low-income countries.

- This is the first published comprehensive comparison of contextualised CEAs for multiple health interventions targeting both non-communicable diseases (NCDs) and maternal, neonatal and child health.
- Scaling up maternal, neonatal and child health interventions, together with primary preventions of cardiovascular diseases, gives higher expected health benefits and lower budget impacts than investments in other NCD interventions.
- The contextualised CEA method here is user-friendly and gives a clear visualisation of the opportunity cost when deciding on national healthcare investments.

## Introduction

Globally, most countries have committed themselves to move towards universal health coverage (UHC) which is a key subtarget of the Sustainable Development Goals for health.^1–3^ UHC has been defined as all people receiving quality health services that meet their needs without being exposed to financial hardship in paying for the services.^4^ Given resource constraints, this does not entail all possible services, but a comprehensive range of essential services that is well aligned with other social goals.^5^ Competing priorities within the health sector and across other sectors necessitates careful work in defining an optimal and feasible path to UHC.

Cost-effectiveness analysis (CEA) identifies interventions that maximise population health, an important objective in resource constrained health systems.^4 6^ Health economic evaluations are now being used in several ongoing national benefit package revisions in low-income countries.^7^ Ranking interventions by cost-effectiveness can be used to explicitly identify the health services that deliver the highest health impact for the lowest cost. While recent academic debates highlight the need to include concerns for fairness issues like financial risk protection^8^ and priority to worse off,^5 9^ close attention to evidence about health maximisation and distribution is important in policy decisions.^5^

More health economists are needed in low-income countries. The majority of health economic evaluations are conducted in high-income countries. Regional CEAs may be the only available evidence in a low-income country. Often, important interventions lack evidence from health economic evaluations and fiscal analyses. Regional CEAs should be translated with caution to country levels, and preferably contextualised and parametrised to the respective country.^10^ Local demography, epidemiology and health system may have large impact on expected costs and health effects. CEAs and results from one setting cannot easily be transferred to another.^11^ One way of overcoming this is to contextualise methods, data and analyses to ensure that models fit and reflect national health system objectives and constraints. Involvement of local health economists or other with similar skills is important in this work.

This paper presents a summary of results from three separate contextualised CEAs targeting maternal, newborn and child health (MNCH), mental and neurological conditions and cardiovascular diseases (CVD) from Ethiopia.^12–14^ In doing this, a total of 61 interventions are rank ordered based on the incremental cost-effectiveness rates (ICERs). We discuss lessons learnt from the initial analytical processes and how this evidence can inform priority setting in Ethiopia.

## Ethiopian context

Ethiopia is among the countries that shows a strong commitment to implement the UHC as part of aiming for the Sustainable Development Goals (SDG) for health.^15^ With a GDP of US$ 772 per capita (2018),^16^ Ethiopia aspires to *‘* *…transition into a lower-middle income country by 2025 and a middle-middle income country by 2035.*’^17^ Aligned with that, the Ministry has conducted an ‘envisioning of the health sector’ towards UHC, where they identified good-performing and poor-performing lower middle-income countries in order to identify possible policy options that Ethiopia can learn from.^17–19^ Ethiopia developed an Essential Package of Health Services in 2005 that is currently under substantial revision.^20^ While Ethiopia has made significant progress in improving access to primary care over the past decades, the coverage remains low for most essential health services at a primary and specialised level (see table 1).^21^ Health economic evaluations and fiscal space analyses of multiple interventions are needed with emerging non-communicable diseases and injuries (NCDI) and a still unfinished agenda for many of the paediatric, obstetric and infectious disease interventions.

**Table 1:** Current coverage of 13 key maternal, child and neonatal health services in Ethiopia^12^

Maternal and neonatal interventions	Baseline coverage (%)
Neonatal resuscitation (institutional)	26
Kangaroo mother care	22
Newborn sepsis—Injectable antibiotics	26
Antibiotics for pPRoM	3
Management of pre-eclampsia and eclampsia	3
Antenatal corticosteroids for preterm labour	0
Induction of labour (beyond 41 weeks)	3
Safe abortion	37
Maternal sepsis case management	22
Active management of the 3rd stage of labour	23
Tetanus toxoid (pregnant women)	49
Syphilis detection and treatment (pregnant women)	31
Calcium supplementation	0

pPRoM, preterm premature rupture of membrane.

Maternal, reproductive, neonatal, child and adolescent health is a cornerstone in the Health Sector Transformation Plan (2015–2020) in Ethiopia.^17^ However, such services are far from universally available, and further scale-up are likely to compete with a range of services for NCDI. Only 28% of all deliveries were conducted by a skilled person according to Ethiopia Demographic and Health Survey (2016).^21^ The launch of the National Mental Health Strategy in 2012 and the National Strategic Action Plan for Prevention and Control of NCDIs in 2014 indicates a higher priority to mental and substance use disorders.^22 23^ A national commission on NCDI in Ethiopia recently launched an essential healthcare package for prioritised NCDI interventions.^24^ A majority of the mental health and neurological and CVD interventions are currently not publicly available, and the costs are therefore covered by the patients and their households in Ethiopia.^25–27^ Taking UHC as an overarching goal, Ethiopia envisages ambitious and progressive scale-up of a comprehensive range of services mainly through primary healthcare and the Health Extension Program.^18^ So far, only a few CEAs are made based on contextualised data from Ethiopia.

## Selecting policy relevant interventions

After consulting with the Ministry of Health officials in Ethiopia on what they found as the collection of the most policy relevant interventions, we assessed cost-effectiveness, targeting three broad disease categories separately: (1) treatment of newborn disorders (sepsis and other infections, respiratory distress, premature births, tetanus, still-births, birth asphyxia and complications during labour) and maternal disorders (sepsis, hypertensive disorders of pregnancy, intrapartum events, and unsafe abortion); (2) treatment of depression, schizophrenia, bipolar disorder and epilepsy and (3) prevention and treatment of myocardial infarction and stroke. The range of interventions assessed was selected through an active participation of key policy makers in the Federal Ministry of Health (FMOH) in view of the envisaged policy direction: (i) relevance to the scale-up of primary healthcare and (b) emerging challenges from NCDIs such as CVD and mental health.

## Calculating best buys

Population based models were used to estimate costs and effectiveness of the selected interventions. WHO-CHOICE regional CEA models were substantially revised with Ethiopian epidemiological, demographic, efficacy and cost data whenever possible. Details of the methods have been reported separately in the respective CEA papers.^12–14^ The currency year in each of the contextualised CEAs are here inflation-adjusted to 2017 values to facilitate comparisons across all interventions. A null scenario with no coverage of services was used as baseline for each cardiovascular, mental and neurological intervention to estimate ICERs. Therefore, the cheapest interventions presented here can actually be dominated since the null scenario of the mutually exclusive interventions are not presented in the tables. Current coverage was used to calculate ICERs of maternal, child and neonatal health services.^12^ Target coverages for all cardiovascular and maternal, child and neonatal health interventions were set to 20%, which was considered a realistic and not too ambitious coverage increase of these interventions in an Ethiopia setting. The existing National Mental Health Strategy in Ethiopia was used as a reference to set target coverage for treatment of depression (30%), bipolar disorder (50%), schizophrenia (75%) and epilepsy (75%).^22^

**Figure 1:**
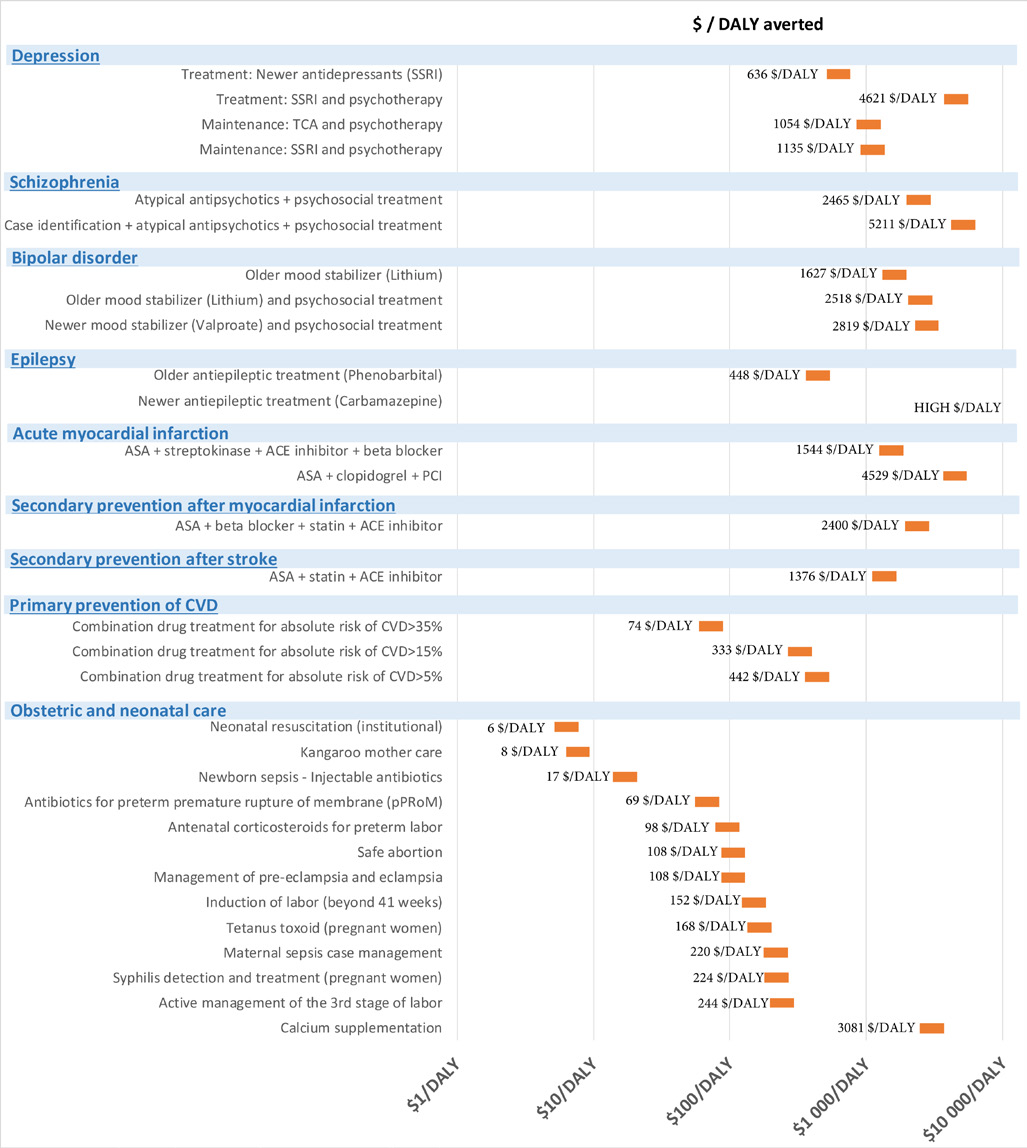
Contextualised cost-effectiveness of a complete set of interventions—ranking of 13 maternal and child health, 19 mental and neurological and 29 cardiovascular interventions by cost-effectiveness.

Figure 1 and table 2 show the results from the three contextualised CEAs. The ICERs for the interventions vary widely with an order of magnitude ranging by up to several hundred folds.

**Table 2:** Contextualised cost-effectiveness of a complete set of interventions—ranking of 13 maternal and child health, 19 mental and neurological and 29 cardiovascular interventions by their respective ICER

Condition	Interventions	Annual cost (US$)*	DALYs averted	ICER (US$/DALY)	Rank
Major depressive disorder	Older antidepressants (TCA)	15 935 000	24 300	D	–
	Newer antidepressants (SSRI)	18 542 000	29 100	636	17
	Psychotherapy	68 138 000	29 100	D	–
	Older antidepressants (TCA) and psychotherapy	69 632 000	34 100	D	–
	Newer antidepressants (SSRI) and psychotherapy	71 402 000	40 600	4621	
Major depressive disorder	Maintenance: Older antidepressants (TCA) and psychotherapy	62 081 000	58 900	1054	18
	Maintenance: Newer antidepressants (SSRI) and psychotherapy	65 789 000	62 200	1135	19
Schizophrenia	Typical antipsychotics	23 639 000	4900	D	–
	Atypical antipsychotics	23 635 000	6000	D	–
	Typical antipsychotics+psychosocial treatment	25 058 000	9000	D	–
	Atypical antipsychotics+psychosocial treatment	26 252 000	10 600	2465	24
	Case id+management: Typical antipsychotics and psychosocial treatment	30 097 000	11 100	D	–
	Case id+management: Atypical antipsychotics and psychosocial treatment	31 290 000	11 600	5211	28
Bipolar affective disorder	Older mood stabiliser (Lithium)	28 560 000	17 600	1627	22
	Older mood stabiliser (Lithium) and psychosocial treatment	33 045 000	19 300	2518	25
	Newer mood stabiliser (Valproate)	31 913 000	18 600	D	–
	Newer mood stabiliser (Valproate) and psychosocial treatment	36 418 000	20 500	2819	26
Epilepsy	Older antiepileptic treatment (Phenobarbital)	30 874 000	68 900	448	16
	Newer antiepileptic treatment (Carbamazepine)	67 170 000	68 900	HIGH	31
Acute ischaemic heart disease	ACE inhibitor	3 082 000	300	D	–
	Beta-blocker	3 084 000	600	D	–
	ASA	3 087 000	1000	D	–
	Streptokinase	3 662 000	1200	D	–
	ASA+clopidogrel	3 094 000	1400	D	–
	ASA+streptokinase	3 692 000	2100	D	–
	ASA+streptokinase+ACE inhibitor+beta-blocker	3 790 000	2100	D	–
	ASA+streptokinase+ACE inhibitor	3 700 000	2400	1544	21
	Primary PCI	10 755 000	2700	D	–
	ASA+clopidogrel+PCI	11 032 000	4000	4529	29
Post-acute IHD	Statin	3 552 000	300	D	–
	Beta-blocker	3 281 000	500	D	–
	ACE inhibitor	3 306 000	500	D	–
	ASA+beta-blocker	3 337 000	700	D	–
	ASA+beta-blocker+statin	3 659 000	1000	D	–
	ASA+beta-blocker+statin+ACE inhibitor	3 736 000	1600	2400	23
Acute ischaemic stroke	ASA	3 282 000	100	52 102	30
Post-acute stroke	ACE inhibitor	3 730 000	900	D	–
	ASA	3 707 000	1000	D	–
	ASA+statin	4 414 000	2400	D	–
	ASA+statin+ACE inhibitor	4 518 000	3300	1376	20
Primary prevention of CVD	Individual cholesterol treatment((tot. chol.>6.2 mmol/L)	6 059 000	8800	D	–
	Individual cholesterol treatment (tot. chol. >5.7 mmol/L)	13 778 000	19 100	D	–
	Individual hypertension treatment (SBP>160 mm Hg)	9 510 000	98 900	D	–
	Combination drug treatment for absolute risk of CVD>35%	9 315 000	125 700	74	5
	Individual hypertension treatment (SBP >140 mm Hg)	25 196 000	125 700	D	–
	Combination drug treatment for absolute risk of CVD>25%	12 753 000	128 000	D	–
	Combination drug treatment for absolute risk of CVD>15%	18 696 000	153 900	333	14
	Combination drug treatment for absolute risk of CVD>5%	34 835 000	190 400	442	15
Neonatal disorders	Neonatal resuscitation (institutional)	353 000	54 700	6	1
Neonatal disorders	Kangaroo mother care	287 000	36 700	8	2
Neonatal disorders	Newborn sepsis—Injectable antibiotics	906 000	52 100	17	3
Maternal/neonatal disorders	Antibiotics for pPRoM	591 000	8500	69	4
Neonatal disorders	Antenatal corticosteroids for preterm labour	837 000	8600	98	6
Maternal disorders	Safe abortion	737 000	6800	108	7
Maternal/neonatal disorders	Management of pre-eclampsia and eclampsia	519 000	4800	108	7
Neonatal disorders	Induction of labour (beyond 41 weeks)	393 000	2600	152	9
Maternal/neonatal disorders	Tetanus toxoid (pregnant women)	2 688 000	16 000	168	10
Maternal disorders	Maternal sepsis case management	1 151 000	5200	220	11
Maternal/neonatal disorders	Syphilis detection and treatment (pregnant women)	1 522 000	6800	224	12
Maternal/neonatal disorders	Active management of the third stage of labour	1 617 000	6600	244	13
Maternal/neonatal disorders	Calcium supplementation	4 949 000	1600	3081	27

*2018 US$.

ACE, angiotensin-converting enzyme; ASA, acetylsalisylic acid; CVD, cardiovascular disease; ICER, incremental cost-effectiveness rate; IHD, ischemic heart disease; PCI, percutaneous coronary intervention; pPRoM, preterm premature rupture of membrane; SBP, systolic blood pressure; SSRI, selective serotonin reuptake inhibitors; TCA, tricyclic antidepressants.

**Figure 2:**
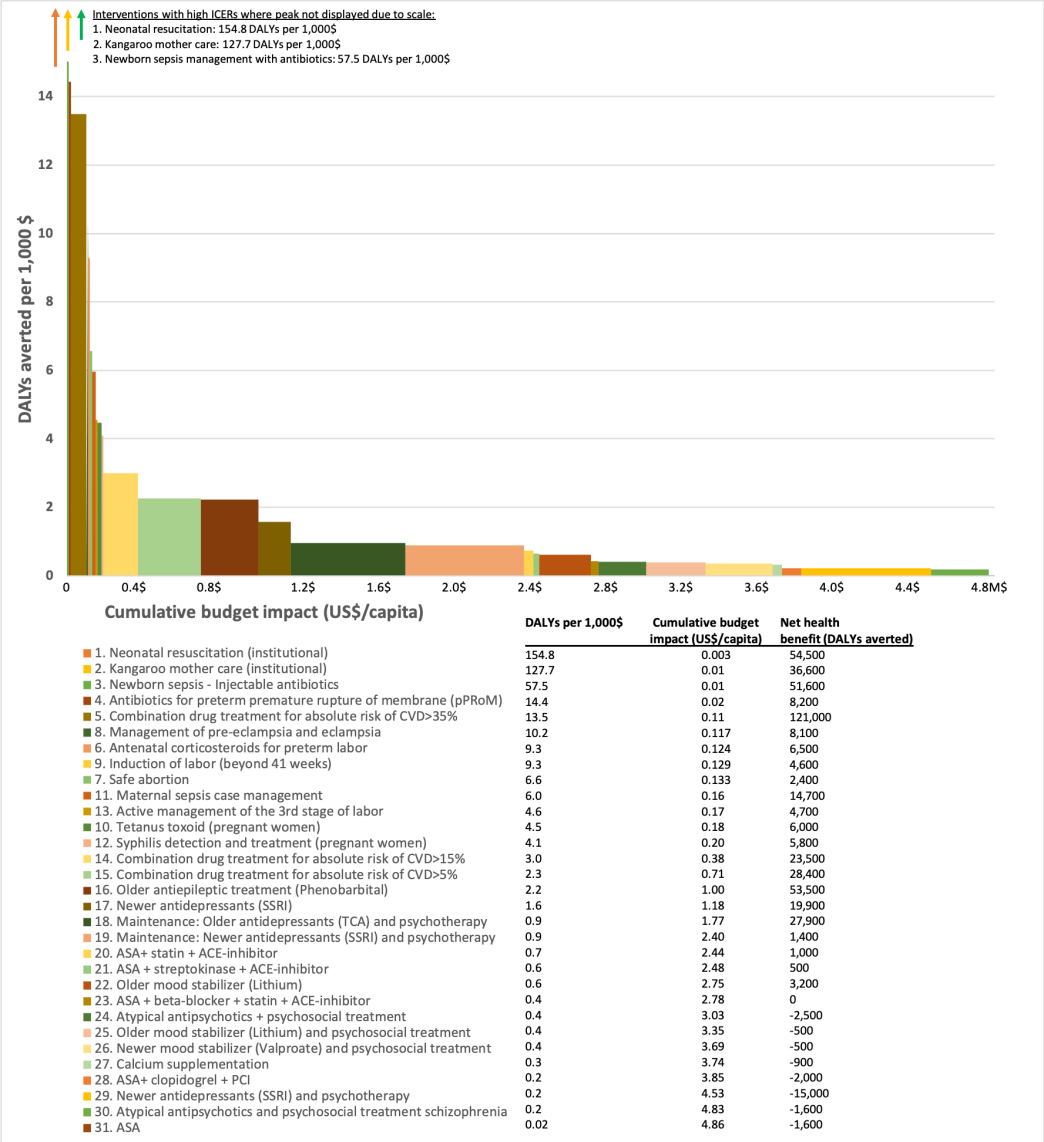
Cost-effectiveness (DALYs averted per $1000), bar heights and cumulative budget impact, bar width, of all 31 interventions that are not dominated, ordered from the lowest (left) to highest (right) cost-effectiveness (numerical values, including net health benefits (with WTP threshold $2000/DALY averted), are shown at bottom).

Figure 2 visualises the budget impact of investing in the 31 non-dominated interventions from table 1. A US$1 increase per capita in the annual Ethiopian health budget, currently at US$28,^16^ could allow inclusion of around half of these interventions into the health system.

Ochalek *et al* argue that information of ICERs are not sufficient for setting priorities between interventions since the size of potential health impacts are not specified with such rates.^7^ They argue that estimates of net DALYs averted best captures potential health impacts, and net health benefit of each intervention is presented at the bottom of figure 2. In our calculations of net health benefits, we set the Ethiopian willingness to pay threshold at US$2000 per DALY averted, acknowledging that this a rough and arbitrary threshold. More in-depth financial costing analysis is needed for better precision. Nine of the interventions (table 2) are expected to give negative or zero net health benefits in return with a US$2000 budget threshold. Priority to interventions with expected negative net health benefits, for example, primary percutaneous coronary intervention (PCI) for myocardial infarction, calcium supplementation during pregnancy and some of the mental health interventions, would thus cause more harm than good for population health in Ethiopia—if the willingness to pay threshold is less than US$2000 per DALY averted.

## Lessons learnt

To the best of our knowledge, this is the first comparison of comprehensive contextualised CEAs for health interventions in an Ethiopia setting. By using standardised and comparable methods and data inputs, we are able to produce a league table allowing reasonable comparability across intervention categories. We describe CEAs as contextualised if they include national analysts and use country-specific input for either costs, epidemiology, demography, baseline coverage or effects. Several of the coauthors are Ethiopians and have experience with policy and planning, and they contributed substantially to the data collection and analysis. This was important for making the analyses policy relevant and locally relevant. In addition, this is important capacity building and a way to train people in Ethiopia in health economic methods. Defining, expanding and financing key services are arguably the most important first steps in the process of translating results from CEAs to actual health policy. Below, we discuss our lessons learnt for each category of interventions in detail and point at the contribution these contextualised CEAs may provide to policy makers as well as its challenges and limitations.

### Lessons: maternal and child health

Almost all of the maternal and neonatal health interventions have low ICERs. The total annual cost of increasing coverage of all maternal and neonatal interventions by 20% is estimated to be around US$21 million (US$0.2 per capita), with an aggregated expected net health benefit of around 204 000 DALYs averted. These services are also stated as high priority services in Ethiopian policy documents. Yet, they have a low effective coverage levels in Ethiopia. In a recent Lancet publication, Ruducha *et al* show how child mortality has decreased substantially in Ethiopia, and now neonatal morality makes up 46% of the under-5-mortality.^28^ For most policy makers, it is well known that maternal and child care services are effective and efficient interventions, while neonatal interventions have received less attention. In Memirie *et al*’s study, we found that introducing neonatal resuscitation, kangaroo mother care and treatment of newborn sepsis with injectable antibiotics in neonatal intensive care units have the lowest ICERs. The ICERs for these interventions are between US$6 and US$17/DALY averted and the expected net health benefits are expected to be 143 700 DALYs averted in total, at a 20% incremental overage level.^12^ In the latest demographic health survey from Ethiopia (2016), the neonatal mortality rate is 29 deaths per 1000 deliveries. However, only 30% of children<6 months with fever seek care at a health facility and 28% of all births are delivered by a skilled obstetrician or midwife or other trained skilled health personnel. While maternal and child health interventions have been, and still are high priority in Ethiopia, most of the NCD interventions analysed here have higher ICERs. There is a risk that a priority to NCDI interventions can diminish the priority to maternal and neonatal interventions and reduce population health levels. Solberg *et al* are some of the many arguing that saving a newborn life has relatively less value than saving older children or adult lives.^29^ If less value is given to save newborn lives, it may be justifiable to diverge from obstetric and neonatal interventions. Contextualised CEAs provide evidence and can visualise such trade-offs explicitly in fair and deliberative decision-making processes.

### Lessons: cardiovascular diseases

Of note is that most of the CVD interventions in figure 1 and table 2 were not included in the 2005 essential health services package.^30^ Many of these are now considered for being included in the package as part of the current revision. Primary prevention of CVD has one of the lowest ICERs of all 61 interventions. In the contextualised CEA by Tadesse *et al*, we found that primary prevention of CVD in primary healthcare, at a health centre and primary hospital level, with an absolute risk-based approach cost between US$74 and US$442 /DALY averted (or between 2.3 and 13.5 DALYs averted per US$1000 invested). ICERs, health benefits and budget impact vary by risk eligibility threshold. A high threshold policy, >35% 10-year risk of CVD, is estimated to give 13.5 DALYs in return per US$1000 invested (US$74/DALY averted), avert 121 000 DALYs and cost US$9.3 million in total. A low risk policy, 5% 10-year risk of CVD, is estimated to give 2.3 DALYs in return per US$1000 invested (US$442/DALY averted), avert 28 000 DALYs and cost US$34.8 million in total. The reason why ICERs increase with lower risk thresholds is that the number of CVD events prevented per person taking antihypertensives and statins diminishes with lower eligibility risk thresholds. Even if primary prevention has low ICERs, it is important to think carefully how this service is best integrated into the health system during a scale-up. The most advanced treatment of myocardial infarction (PCI and clopidogrel) has an ICER at US$4529 per DALY averted (0.2 DALYs averted per US$1000 invested) and negative net health benefits (−2000 DALYs averted), at a 20% incremental coverage level and a budget threshold of US$2000/DALYs. Streptokinase is slightly less efficacious than PCI, but has a much lower ICER (US$1544/DALY averted or 0.6 DALYs averted per US$1000 invested) and a net benefit at around 500 DALYs averted in an Ethiopian setting.

The health system in Ethiopia has up to now given high priority to interventions targeting communicable (eg, HIV, Tb and malaria) and MNC conditions. Primary prevention of CVD, and treatment of other chronic conditions, depends on long-term patient-centred healthcare, trained personnel and well-functioning referral systems.^31^ Health-information systems, sustainable delivery of drugs and regulatory capacities to manage private actors is also important in the implementation of primary prevention into the health system in Ethiopia. These are key challenges that policy makers need to handle when deciding whether primary prevention should be scaled-up in a country where the majority of the population live in rural areas. If only the interventions for CVD with the lowest ICERs, within the CVD category, were scaled-up by 20% in Ethiopia (primary prevention of CVD for individuals with >35% risk of CVD; ASA, streptokinase and ACE-inhibitor for treatment of acute myocardial infarction; ASA, beta-blocker, statin and ACE-inhibitor as secondary prevention after myocardial infarction and ASA, statin and ACE-inhibitor as secondary prevention after stroke), total annual health benefit is expected to be 122 600 DALYs averted and budget impact is estimated to be around US$21.3 million (US$0.20 per capita).

### Lessons: mental health

The mental health interventions have the highest ICERs of the ones we considered, and these interventions are seen, with low and wide bars, in the right corner of figure 2. In the CEA by Strand *et al*,^13^ we found that treatment of depression with antidepressants (SSRI) cost US$636/DALY averted (1.6 DALYs averted per US$1000 invested) and is expected to avert 19 900 DALYs in total. The combination 18 hours of psychotherapy and older antidepressants (TCA) for preventing relapse of depression, gives an ICER at US$1134/DALY averted (0.9 DALYs averted per US$1000 invested) and a net health benefit of 27 900 DALYs averted. Treatment of epilepsy with phenobarbital cost US$448/DALY averted (2.2 DALYs averted per US$1000 invested) and a net health benefit of 53 500 DALYs averted. Previously, we have applied a methodology of Extended Cost-Effectiveness Analysis to this CEA of mental and neurological healthcare in Ethiopia. Around 80% of the investments for scaling-up treatment and maintenance of depression was expected to be returned in the form of productivity gains.^26^ Treatments of disorders such as schizophrenia and bipolar disorders have higher ICERs and lower expected health benefits, but prioritising such services could be justified by assigning higher priority to more severe diseases.^5^ Whether it is acceptable to introduce interventions with high ICERs due to other fairness concerns like severity of disease are important for policymakers to discuss and decide on. If so, the cost-effectiveness threshold for less severe diseases must be lower. If only the most cost-effective interventions for each of the mental and neurological conditions were scaled-up by 30%–75% in Ethiopia (SSRI and psychotherapy for depression; lithium for bipolar disorder; risperidone for schizophrenia and phenobarbital for epilepsy), total annual cost is estimated to be around US$156 million (US$1.5 per capita) and the total health benefit expected to be 102 000 DALYs averted.

## Contextualisation processes: thresholds and building health economic capacity

In Ethiopia, as in most other countries, there is a strong pressure to implement interventions which are not cost-effective. Such investments can level down rather than level up population health by displacing alternative health interventions. One example is dialysis treatment for end-stage kidney diseases. While the ICER of this intervention is far higher than the ICERs presented in our study, many African countries are now providing dialysis treatment in an increasing scale.^32 33^

Following suggestions by Claxton, Woods and others,^34 35^ we believe previous cost-effectiveness thresholds were not well-founded on empirical grounds and may not indicate the actual opportunity costs of decisions.^36^ New interventions can only be financed within existing budget constraints. Cost-effectiveness thresholds therefore become important in healthcare priority setting. Beneficial interventions are at risk of being displaced if less cost-effective interventions are introduced into the healthcare system without additional funding. As a consequence, less population health may be achieved if cost-effectiveness thresholds are too high. A cost-effectiveness threshold is not suggested here, but we use a threshold of US$2000 per DALYs averted in the net health benefit analysis as an example. Budget threshold decisions are up to policy makers to decide on. Such threshold discussions are important in order to succeed with a gradual and realistic scale-up of high priority services in the years to come. By rank ordering all these 61 interventions according ICERs, and excluding dominated interventions, and by presenting the expected net health benefits and budget impact of these interventions, we hope to provide policymakers accessible evidence that can be used to assess willingness to pay for these essential healthcare services.

In these analyses, suboptimal target coverage levels are used. Target coverages were set based on discussion with experts from the different national health programmes in Ethiopia and all policy makers preferred analyses based on what they saw as feasible and realistic target coverage levels. This deviates from the WHO-CHOICE recommendation to estimate cost per health gain if interventions are implemented at full scale. Since ICERs in the models were not sensitive to target coverage levels (multiple levels were tested), we decided to adhere to demands of policy makers of applying what they saw as feasible targets.

Although our analysis is not exhaustive of all possible health interventions, we have demonstrated the importance of generating policy relevant and contextualised evidence. One way of facilitating systematised and explicit priority setting is to train policy makers in doing CEAs with contextualised data and visualisation of results. Long-term investments in training and close collaborations have been crucial in the development of the three studies presented here. Now, the Ethiopian coauthors of these CEAs provide technical support in national policy priority setting processes and independently conduct local CEAs. In addition, they are key in establishing a new Addis Ababa Centre for Ethics and Priority Setting (ACEPS) that aim to train decision makers in neighbouring African countries to conduct health economic evaluations and systematic priority setting.

## Conclusion

A comprehensive ranking of interventions in league tables provides comparability across categories of interventions and gives a clear presentation of evidence and opportunity cost. In systematic priority setting, such evidence should be considered together with concerns for equity and financial risk protection. An annual US$2.8 investment per capita in 22 interventions in Ethiopia can increase coverage by 20%–75% and around 0.5 million net DALYs averted is expected in return in total per year—with a willingness to pay threshold at US$2000 per DALY averted. Capacity building is an important element in resource-constrained settings, as the demand for health economic evaluations are immensely high.
